# Topographical curvature is sufficient to control epithelium elongation

**DOI:** 10.1038/s41598-020-70907-0

**Published:** 2020-09-08

**Authors:** Pablo Rougerie, Laurent Pieuchot, Rafaela Silva dos Santos, Julie Marteau, Maxence Bigerelle, Pierre-François Chauvy, Marcos Farina, Karine Anselme

**Affiliations:** 1 Laboratório de Biomineralização, Centro de Ciênça da Saúde, Instituto De Ciências Biomédicas, Federal University of Rio de Janeiro, Rio de Janeiro, 21941-902 Brazil; 2 Université de Haute-Alsace, CNRS, IS2MUMR 7361, 68100 Mulhouse, France; 3 Instituto de Biofisica Carlos Chagas Filho, Centro de Ciências da Saúde, Federal University of Rio de Janeiro, Rio de Janeiro, 21941-902 Brazil; 4 Université de Valenciennes et du Hainaut Cambrésis, LAMIHUMR-CNRS 8201, Le Mont Houy, 59313 Valenciennes, France; 5 France.Micropat SA, Côtes-de-Montbenon 30, 1003 Lausanne, Switzerland

**Keywords:** Biomaterials, Tissue engineering, Cell biology, Morphogenesis

## Abstract

How biophysical cues can control tissue morphogenesis is a central question in biology and for the development of efficient tissue engineering strategies. Recent data suggest that specific topographies such as grooves and ridges can trigger anisotropic tissue growth. However, the specific contribution of biologically relevant topographical features such as cell-scale curvature is still unclear. Here we engineer a series of grooves and ridges model topographies exhibiting specific curvature at the ridge/groove junctions and monitored the growth of epithelial colonies on these surfaces. We observe a striking proportionality between the maximum convex curvature of the ridges and the elongation of the epithelium. This is accompanied by the anisotropic distribution of F-actin and nuclei with partial exclusion of both in convex regions as well as the curvature-dependent reorientation of pluricellular protrusions and mitotic spindles. This demonstrates that curvature itself is sufficient to trigger and modulate the oriented growth of epithelia through the formation of convex “topographical barriers” and establishes curvature as a powerful tuning parameter for tissue engineering and biomimetic biomaterial design.

## Introduction

Understanding the mechanisms controlling and guiding the growth of tissues is of fundamental importance. In particular, the ability of epithelia to break isotropy and grow preferentially along a specific direction is a key part of embryo development and wound healing. Unsurprisingly, it is also an important part of tissue engineering efforts aiming at fostering tissue repair or growing in vitro organoids and replacement tissues.

Over the years, the microenvironment geometry has emerged as a relevant parameter of tissue morphogenesis^1^. Indeed, cells tend to align and migrate along the main orientation of anisotropic surfaces, a process termed “contact guidance”^2,3^. Parallel grooves and ridges are among the most common model surfaces for contact guidance. Over such surfaces, single cells elongate and align with the longitudinal axis of the grooves and ridges while epithelial monolayers exhibit an oriented collective migration^4–8^. These topographies are characterized by the presence of angular edges and corners at the junction between a ridge and the adjacent grooves. These so-called “topographical discontinuities” has long been considered instrumental for contact guidance and the resulting anisotropic elongation of cells and epithelia^2,9^. For instance, single cells tend to extend protrusions along such discontinuities and accumulate F-actin or Vinculin there^10,11^. However, the in vivo microenvironment associated with epithelia is edge-less, displaying a complex architecture with fibrillary or curved shapes both at nanometric, cellular, and supracellular scales. The contact-guided epithelial growth could thus be an artefact from in vitro surfaces with topographical discontinuities. It is therefore crucial to determine whether and how the notion of contact-guided epithelium morphogenesis can be extended to edge-less, curved topographies.

Substrate curvature affects the local trajectory and the overall velocity of cells^12–14^. Convexity seems to be associated with higher myosin II-based contractility and lower F-actin contacts, as opposed to concavity^15,16^. Negative curvatures (concavity) also seem to induce detachment or tearing of fully-grown, static and contractile epithelial monolayers^17^. However, the available studies so far focused on single cells migrating over isotropically curved surfaces^12,15^, or on full-grown epithelial monolayers confined around cylindrical wires or inside hollow tubes^13,14^. Importantly, cylindrical surfaces necessarily cause a lateral confinement that restrains the direction of growth of the epithelium along the cylinder axis. In addition, lateral confinement induces changes in the collective migration velocity, independently of any curvature effect^13^. A recent article interestingly looked at the growth of non-confined endothelia over anisotropic sinusoidal surfaces^18^. The results suggest that endothelia grow anisotropically over wavy surfaces. However, it remains unclear how the local curvature affects the growth dynamics of the tissue or the intracellular organization of majorcell components such as the cytoskeleton and nuclei. In addition, this studies compared sinusoidal surfaces with different wavelength, which modifies two key topographical parameters at the same time: the maximal curvature and the period of the convex and concave regions. Given that the period also affects the efficiency of topographical guidance^19,20^, it is therefore crucial to uncouple the curvature and the period in order to be able to draw definitive conclusions on the specific role of curvature itself during tissue growth and morphogenesis. To do so, we engineered anisotropic surfaces made of cell-scale grooves and ridges of different curvature at the ridge/grooves junctions but of similar depth and periods. We then set out to applied these new topographies to assess how these landscape would influence the morphogenesis of growing epithelia. We confirmed that topographical discontinuities are dispensable for contact guidance and we reported that the degree of epithelium elongation is linearly proportional to the level of convexity to which it is exposed. We showed that convex regions act as topographical barriers that control the organization of the actin cytoskeleton and nuclei and impact key processes such as collective migration and oriented cell division. Altogether, this work identifies curvature as a powerful physical cue that can control epithelium morphogenesis, providing new leads for the development of tissue engineering strategies.

## Results

### Cell-scale curvature linearly controls the elongation of growing epithelia

**Figure 1:**
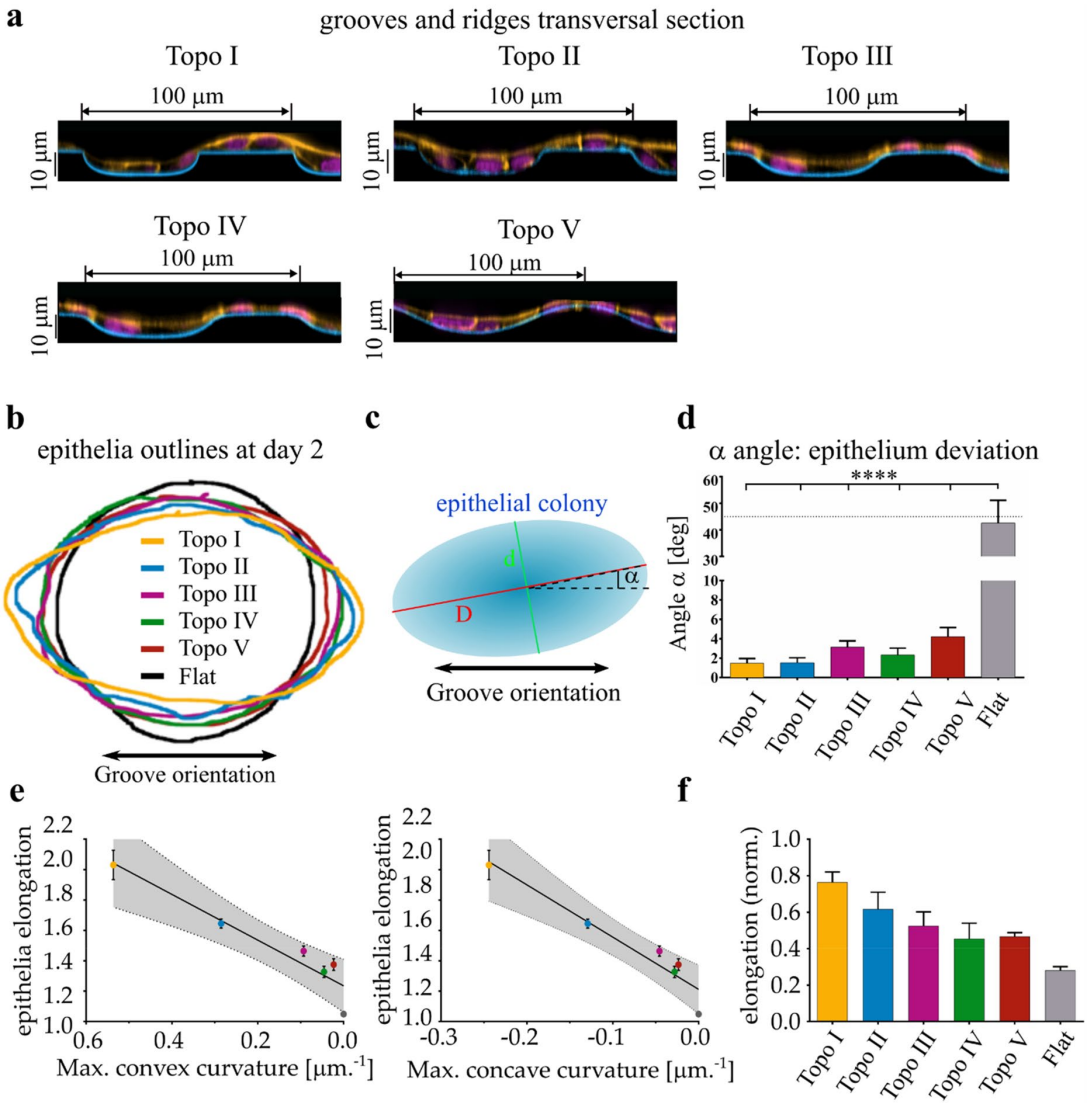
The periodic curvature of the substrate linearly controls the epithelium elongation. (**a**) transversal cross-section of topographies I to V (orange: F-actin, magenta: nuclei, cyan: fibronectin). Note the decreasing curvature at the ridge/groove junctions. Note that cells can fit entirely on the ridges or grooves. (**b**) Representative outlines of epithelial monolayers after 48 h of growth on the surfaces shown in A. The surface longitudinal axis is oriented horizontally. The size of the outlines has been normalized to allow easier comparison of the shapes. (**c**) Schematic of the morphological parameters quantified. In blue, the ellipsoidal epithelial colony after 2 days of growth. The colony alignment with the topography is calculated as the α angle between the grooves and the longer axis of the colony. The colony elongation of the colony is the Aspect Ratio of the colony longer axis D to the shorter axis d. (**d**) Epithelial colony alignment with the topography as explained in C (mean + SEM). Five to 9 independent colonies per topography. The colony orientations on the 5 topographies are all statistically significant compared to flat (a one-way ANOVA followed by Tukey multiple comparison tests showed a statistical difference between the topographies and the flat control, P < 0.0001). (**e**) Epithelial colony elongation after two days of growth as a function of the maximal convex (left) or concave (right) curvature of each surface (see Supplementary Fig. S1 and Experimental Section for curvature calculation). Five to 9 independent colonies (mean + SEM). Linear regression: Elongation = 1.505 × Convex + 1.235, R^2^ = 0.90 1; Elongation = − 3.441 × Concave + 1.212, R^2^ = 0.9124. Dotted lines: 95% confidence interval of the linear regressions. (**f**) Epithelial colony elongation after 2 days of growth as shown in (**e**) normalized by the growth of the colony. The growth is defined by (*A* − *A*_0_)/*A*_0_ with *A* being the colony area at day 2 and *A*_0_ the colony area at day 0.

We engineered five grooves and ridges topographies with a progressive smoothing of the junction between the ridge and the adjacent groove (Topo I–V) (Fig. 1A). Nanoscale grooves and ridges are associated with phenomena of bridging over the grooves owing to the inherent rigidity of cells, hence restricting adhesion formation to the upper regions^4,6,21^. To avoid such an effect that might interfere with the reading of surface shape per se, we focused on cell-scale geometries. Our surfaces have a period of 100 μm and a depth of 10 μm. With this dimensions, cells can fit entirely over the ridges or in the grooves without restriction of access (Fig. 1A). In addition, we had previously shown that peak and valley topographies with 100 μm period induce a strongest cell response in a human mesenchymal stem cells model^22^. Importantly, the period and depth remain unchanged, so the spatial density of the topographical pattern of interest (curved regions) is similar between topographies and the curvature is the main topographical variable. The maximal convex curvature shows a 20-fold drop from the quasi-angular crenellation of Topo I to the perfectly sinusoidal Topo V while the maximal concave curvature exhibits a more moderate tenfold decrease (Supplementary Fig. S1). Of note, Topo I and Topo II are strongly similar in overall shape and ridge width, however the curvature is noticeably different with a two-fold difference of convex curvature radius (Supplementary Fig. S1). The points of highest convex curvature are localized at the side of the ridge on Topo I, II, III, IV, as shown in the color-coded transversal section of the surfaces in Supplementary Fig. S1D. On topo V, the convex regions merge together into a large apical area of low convex curvature).

We monitored the growth of millimetric circular MDCK colonies over fibronectin-coated polydimethylsiloxane (PDMS) surfaces structured with theses topographies. The canine kidney epithelial cell line MDCK was chosen because it is a classic model system extensively used for epithelial growth, morphogenesis and mechanobiology. The maximal possible growth was reached after 48 h. Beyond that time point, epithelial colonies started to grow outside the topographically structured region of the substrate. Colonies growing over flat, isotropic control surface showed an isotropic growth illustrated by their persistent circular shape. By contrast, all the colonies cultured over the edgeless topographies grew into an elliptic shape (Fig. 1B). The average deviation angle from the topography α indicated that epithelial colonies align with the longitudinal axis of the ridges in all curvature condition tested (Fig. 1C,D). As summarized in Table 1, the alignment with the longitudinal axis is improved by more than 40° (deviation angle between 1.5° and 3.6° whereas it reaches 45° on the Flat control reflecting the uniform distribution of the deviation angle in this case). This confirms that epithelia retain their ability of contact guidance even in the absence of topographical discontinuities. Importantly, we noticed that the elongation of the colony (aspect ratio) shows a linear correlation with the maximum convex or concave curvature (Fig. 1B,E). A transversal convex curvature radius of 44 µm increase the average elongation by 30% compared to the Flat control whereas a high curvature (curvature radius of 2 µm) double the elongation of the growing monolayer compared to the control (Table 1). In order to account for a possible effect of the topography on the extent of growth of the monolayer, we also computed the ratio of elongation to growth. We observed that the relationship between topography and elongation was maintained (Fig. 1F). In our approach, we used surfaces of similar period and depth, ensuring a comparable spatial density of topographical signals. Although the width of the flat section of the ridge varies, it is similar between Topo I and Topo II and yet, the change in maximum curvature still correlates with a noticeable drop in colony elongation. Taken together, these observations clearly demonstrate that cell-scale curvature is per se an important morphogenetic cue able to guide and tune the growth of epithelia.

**Table 1:** Summary of morphological changes of the epithelial colonies.

Topography	Max. convex curvature (μm^−1^)	Min. convex radius (μm)	Max concave curvature (μm^−1^)	Min concave radius (μm^−1^)	Average elongation (fold of Flat)	Average alignment (°)
Topo I	0.537	2.0	− 0.244	4.3	1.9	1.8
Topo II	0.285	3.8	− 0.129	8.2	1.6	1.5
Topo III	0.093	11.3	− 0.046	23.6	1.4	3.1
Topo IV	0.046	22.8	− 0.028	37.0	1.3	2.3
Topo V	0.023	44.4	− 0.024	43.3	1.3	3.6
Flat	0	Infinity	0	Infinity	1	45.3

The Values for maximal convex or concave curvature or radii of curvatures are the mean of the values calculated as detailed in the “Material and methods” and in Supplementary Fig. S1.

### Growing epithelia exhibit a progressive restriction of their transversal growth

**Figure 2:**
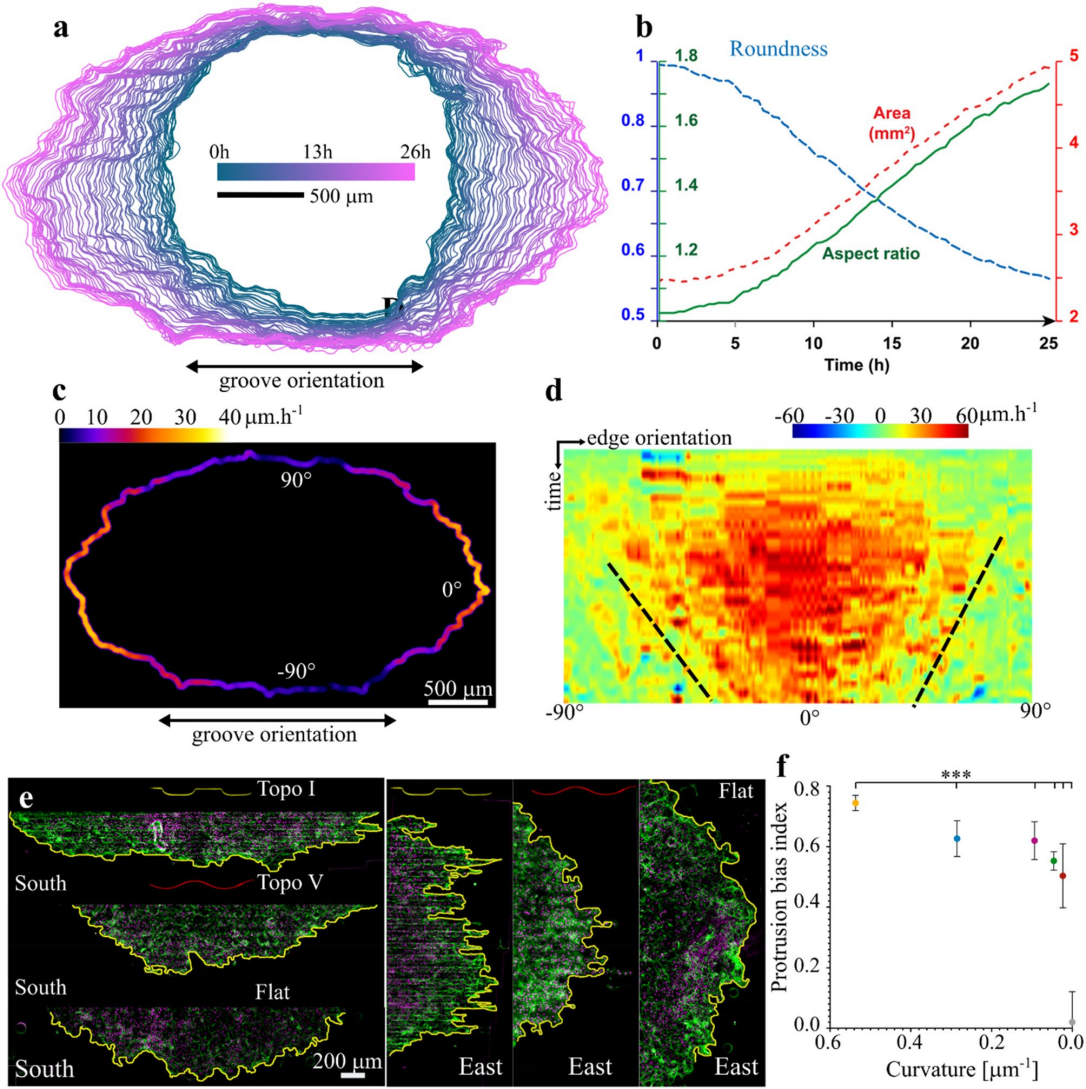
The elongating epithelium shows a progressive hampering of its transversal growth. (**a**) Superposed outlines of a growing MDCK colony on Topo I corresponding to Movie 1. Time lapse: 20 min. (**b**) Longitudinal elongation of a representative MDCK colony over time. Circularity, calculated as $\frac{4\pi \times Area}{{(Perimeter)}^{2}}$ decreases as colonies becomes less circular. Aspect ratio, calculated as the ratio of the longer axis of the bounding ellipse to the shorter axis indicates the stretching of the colony. (**c**) Average local velocity the colony edge outgrowth from (**a**), color-coded and represented on the final colony outline. (**d**) Velocity map of the progression of the colony edge from (**a**). The x axis corresponds to the local edge orientation relatively to the surface longitudinal axis. Zero degree represents the part of the edge extending in the longitudinal direction whereas 90° represent the edge extending transversally across the grooves and ridges. The black dotted lines highlight the progressing restriction of high velocity outgrowth to the edge segments extending longitudinally. (**e**) Representative transversally extending (left) and longitudinally extending (right) epithelium borders used for the calculation of the Protrusion Bias Index; green: actin, magenta: nuclei, yellow: colony border. Note the contrast of tortuosity between both borders on a given topography and between topographies. (**f**) Protrusion Bias Index as a function of convex curvature: a higher value indicates higher contrast in pluricellular protrusion formation between the colony border extending longitudinally and transversally (see Supplementary Note S1 for details). Four to 6 independent colonies for each topography (mean + SEM). A one-way ANOVA showed a statistically difference between all the topography and the Flat control, P < 0.001.

While previous studies reported the effect of anisotropic surfaces on epithelia, their impact on the dynamics of epithelial growth remained unclear. Therefore, we used our topography inducing the strongest phenotype to look deeper into how these surfaces affected the dynamics of epithelial. Over 24 h, colony size and elongation increase simultaneously and linearly (Fig. 2A,B and Supplementary Movie 1). The quantification of the colony border velocity showed that the transversal growth is very slow, with an average speed below 10 μm h^−1^ while the longitudinal growth reaches up to 40 μm h^−1^ on average (Fig. 2C). Therefore, we produced a detailed map of edge velocity as a function of time and of the local border orientation relatively to the topography axis (Fig. 2D). Interestingly it highlighted a progressive restriction of the fast extending regions from a [− 60°; + 60°] arc centered around the surface longitudinal axis to a [− 25°; + 25°] arc 20 h later. Given that the topographies are made of periodic topographical elements (a ridge and a groove), it is possible that the progressive restriction of fast extending regions to the longitudinal direction is due to the repetitive encounter of transversally extending regions with evenly spaced topographical obstacles. Such a “summation” of topographical cues in periodic surfaces had been suggested before^2^. Supporting that idea, we found that longitudinally expanding segment of the monolayer boundary presented clear pluricellular protrusions. (Fig. 2E) These pluricellular protrusions are site of strong cell-ECM traction forces and are considered important in defining the overall direction of collective migration of the monolayer^23,24^. By contrast, the outline of the transversally oriented regions of the monolayer are straighter and apparently devoid of such pluricellular protrusions. This contrast is striking on the most curved surface (Topo I) and gets weaker as the local curvature of the ridge/groove junctions decreases (From Topo I to Topo V). We quantified this anisotropy in pluricellular protrusion formations by developing a Protrusion Bias Index (see “Material and methods” and Supplementary Fig. S2) equal to 0 in the absence of anisotropy and increasing with the contrast in protrusion formation between longitudinal and transversal segments of the monolayer outline. As shown in Fig. 2F, the index is close to 0 on the flat control reflecting the topographical isotropy of this surface, while it is positive on all topographies and decrease along with the curvature. We concluded that the curved regions, extended longitudinally, work as curvature-dependent topographical barriers that repeatedly hamper the formation of pluricellular protrusions and the transversal growth of the monolayer. Under this hypothesis, it becomes clear why it is important to uncouple the curvature parameter from the period parameter as the former would affect the “strength” of those barriers whereas the latter would affect the rate of encounter.

### F-actin and nuclei organizations are sensitive to the magnitude of convex curvatures

**Figure 3:**
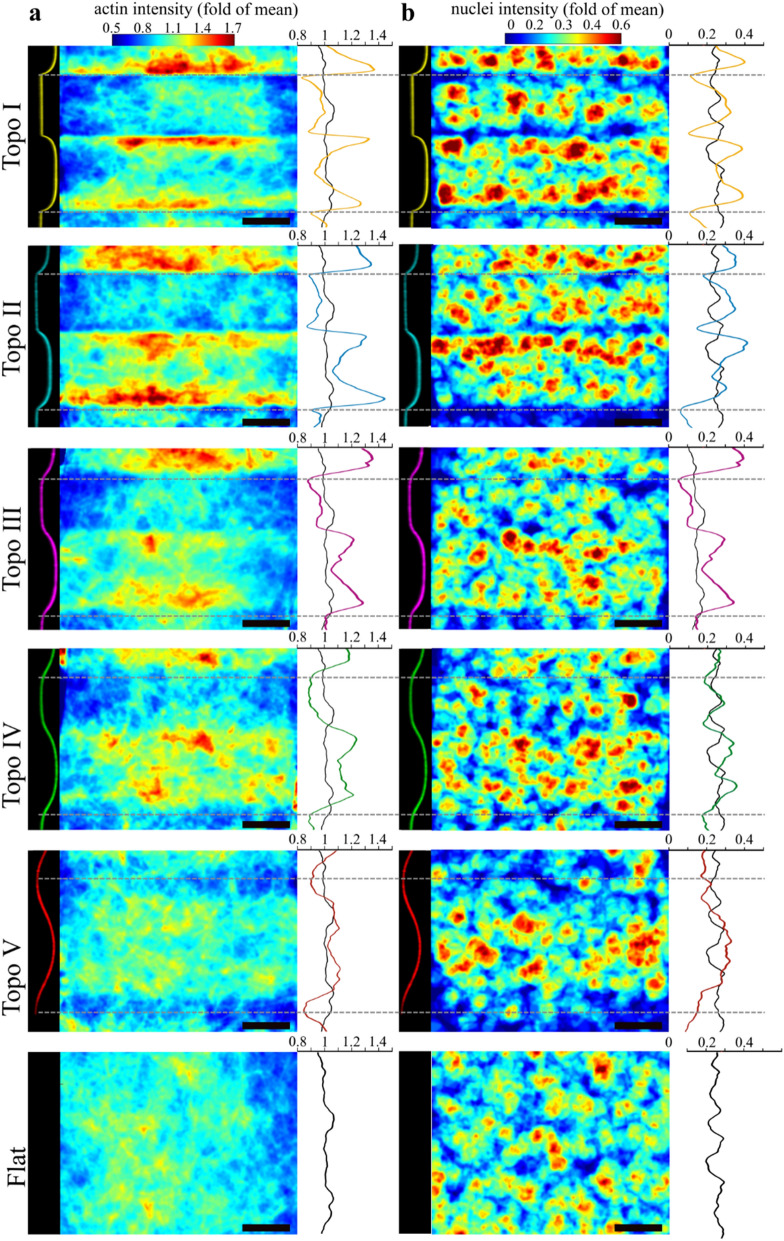
Heterogeneous and anisotropic distribution of F-actin and nuclei in presence of curvature. (**a**) F-actin density map. Blue indicates low density, Red indicates high density. Black dotted line: positions of the points of maximal convex curvature. On the right: the average intensity of the map at every transversal position. Twenty to 30 superposed independent fields were used to generate the maps. Scale bar: 40 μm. (**b**) Nuclei density map. Blue indicates low density, Red indicates high density. Black dotted line: positions of the points of maximal convex curvature. On the right: the average intensity of the map at every transversal position. Twenty to 30 superposed independent fields were used to generate the maps. Scale bar: 40 μm.

The previous observations support the idea that cell-scale curvature is a topographical obstacle that hampers transversal monolayer extension. Nuclei and actin cytoskeleton have been shown to play essential roles in the cell response to curvature, we thus decided to investigate whether they were likely to be involved in the curvature-induced elongation of epithelia^12,14^. We observed that both the F-actin and nuclei show heterogeneous and anisotropic distributions in presence of periodic surface curvature. The F-actin as well as the nuclei densities in the xy plane clearly indicate a heterogeneity with longitudinal stretch of high and low density of F-actin or nuclei (Fig. 3A,B). Importantly, this anisotropic distribution of F-actin and nuclei is less conspicuous as the periodic curvature of the surface decreases. Therefore, the anisotropy of the topography is associated with a corresponding and curvature-dependent anisotropy of F-actin orientation and nuclei distribution.

**Figure 4:**
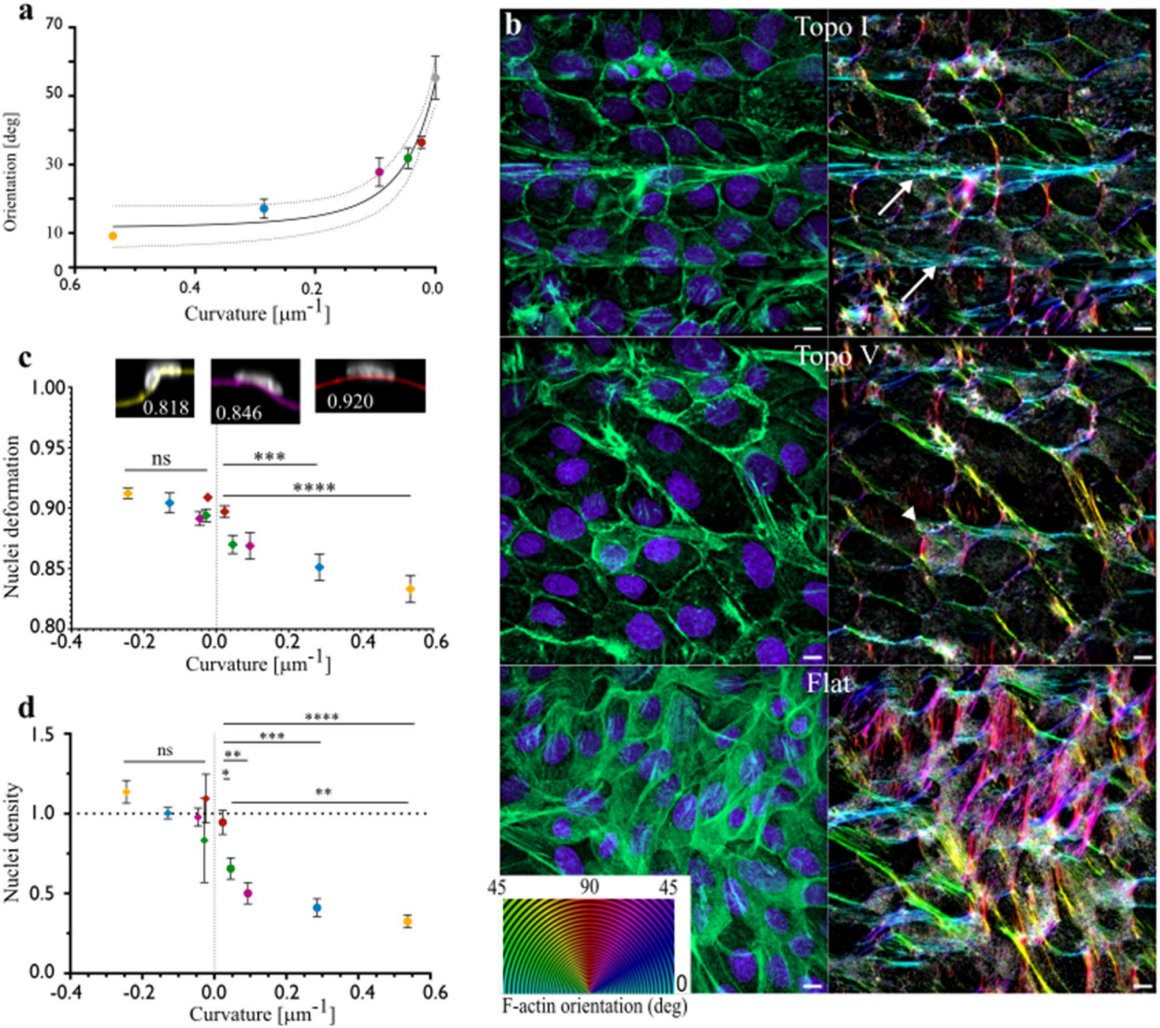
The curvature of the substrate controls the orientation of F-actin and the distribution of nuclei within the monolayer. (**a**) Average F-actin orientation in the xy plane (mean + SEM). Ten fields per colony and 2–7 independent colonies per topography. An angle of 0 indicates a perfect alignment with the topography. The points fit an exponential function *y* = *a* + *b*^*−cx*^ (R^2^ = 0.7726), dotted lines: 95% confidence interval. (**b**) Color coding for F-actin orientation relatively to the surface longitudinal axis for epithelia growing over the most curved (Topo I), least curved (Topo V) and Flat control. The color code is indicated in the bottom left. (**c**) Deformation of the nuclei overlapping the points of maximal convex or concave (mean + SEM). The deformation is assessed by the solidity of the transversal cross-section of the nuclei (ratio between the area of the nucleus transversal cross-section and the area of its bounding ellipse). Lower solidity indicates lower nucleus deformation. Shapes of representative nuclei and their solidity are shown. Thirteen to 30 nuclei per condition. One-way ANOVA followed by Tukey’s multiple comparison test, ****P < 0.0001, ***P < 0.001. (**d**) Proportion of nuclei positioned over the point of maximal convex or concave curvature of a given topography relatively to flat sections (mean + SEM, 6 fields per colony, 2–6 independent colonies per topography). One-way ANOVA followed by Tukey’s multiple comparisons test *P < 0.05, **P < 0.01, ***P < 0.001, ****P < 0.0001.

Supporting the notion that curvature is a topographical obstacle, the convex regions seem to particularly affect the distribution of F-actin and nuclei as their densities drops at the most convex points and accumulate in its vicinity (Fig. 3A,B). This effect is especially clear on the most curved topographies Topo I to Topo III, and becomes less visible on Topo IV and Topo V. We quantified the average spatial orientation of actin fibers in confocal z-stack using OrientationJ Fiji plugin^25^ and we observed that the anisotropic density of F-actin was accompanied with a curvature-dependent longitudinal alignment of the F-actin cytoskeleton (Fig. 4A,B). In particular, 3D reconstruction showed that the high actin density regions close to the most convex point of the surface was made of thick F-actin bundles running longitudinally without crossing the convexity (Fig. 4B, Supplementary Fig. S3 and Supplementary Movies 2 and 3). This suggests that convex regions are not suitable for actin crossing and therefore guide the monolayer cytoskeleton in the longitudinal direction in a curvature-dependent manner.

Regarding the nuclei distribution, we had previously shown in single cells spread over isotropically curved surfaces that nuclei slide away from convex regions as a result of mechanical stress and that this was instrumental for single cell sensing of curvature^12^. To check whether nuclei from our epithelial monolayers were subjected to mechanical stress when contacting the convex regions of the substrates, we used nuclei deformation as a proxy. We measured the solidity of the transversal shape of nuclei positioned over the convex regions (Fig. 4C). A non-deformed ellipsoid nucleus would have solidity equal to one. Our results clearly indicate a curvature-dependent deformation of nuclei when position over the convex regions, whereas concave curvature did not have any clear effect. Consistently, the ratio of nuclei overlapping the sites of maximal convex curvature relatively to flat sections remained below one and increased proportionally to the curvature (from 0.3 on Topo I to 0.9 on Topo V, Fig. 4D). By contrast, the corresponding “concave to flat” ratio did not indicate significant enrichment or exclusion from the concave regions relatively to flat sections. Similarly to actin, albeit with a much milder effect, we observe a trend of increasing nuclei alignment with the longitudinal axis on surfaces with higher curvature (Supplementary Fig. S4A). By contrast, we did not observe a correlation between the type of topography and other morphological aspects of nuclei such as the volume, the elongation or the flatness, with the exception of nuclei being flatter on a totally flat substrate (Supplementary Fig. S4B–D).

Altogether, these results clearly indicate that both F-actin and nuclei, key component of the cell mechanical machinery, are distributed according to the underlying anisotropy of the substrate and that this distribution is proportional to the periodic convex curvature of the substrate. Therefore, they are sensitive to curvature and it strongly suggests that they both participate in the curvature reading by the monolayer and the resulting contact-guided oriented growth. Consistently with what we had previously demonstrated in single cells over isotropic surfaces, convexity appears to be a mechanically unfavorable topographical feature from which F-actin and nuclei are partially excluded. Within the context of anisotropic curvature such as ours, longitudinal stretches of convex regions thus create curvature-dependent “topographical barriers” unsuitable for nuclei crossing and F-actin positioning. This may in turn guide the cytoskeleton and cell orientation along the longitudinal axis of the surface and induce the oriented growth of the tissue.

### Periodic curvature induce a curvature-dependent guidance of key morphogenetic mechanisms

**Figure 5:**
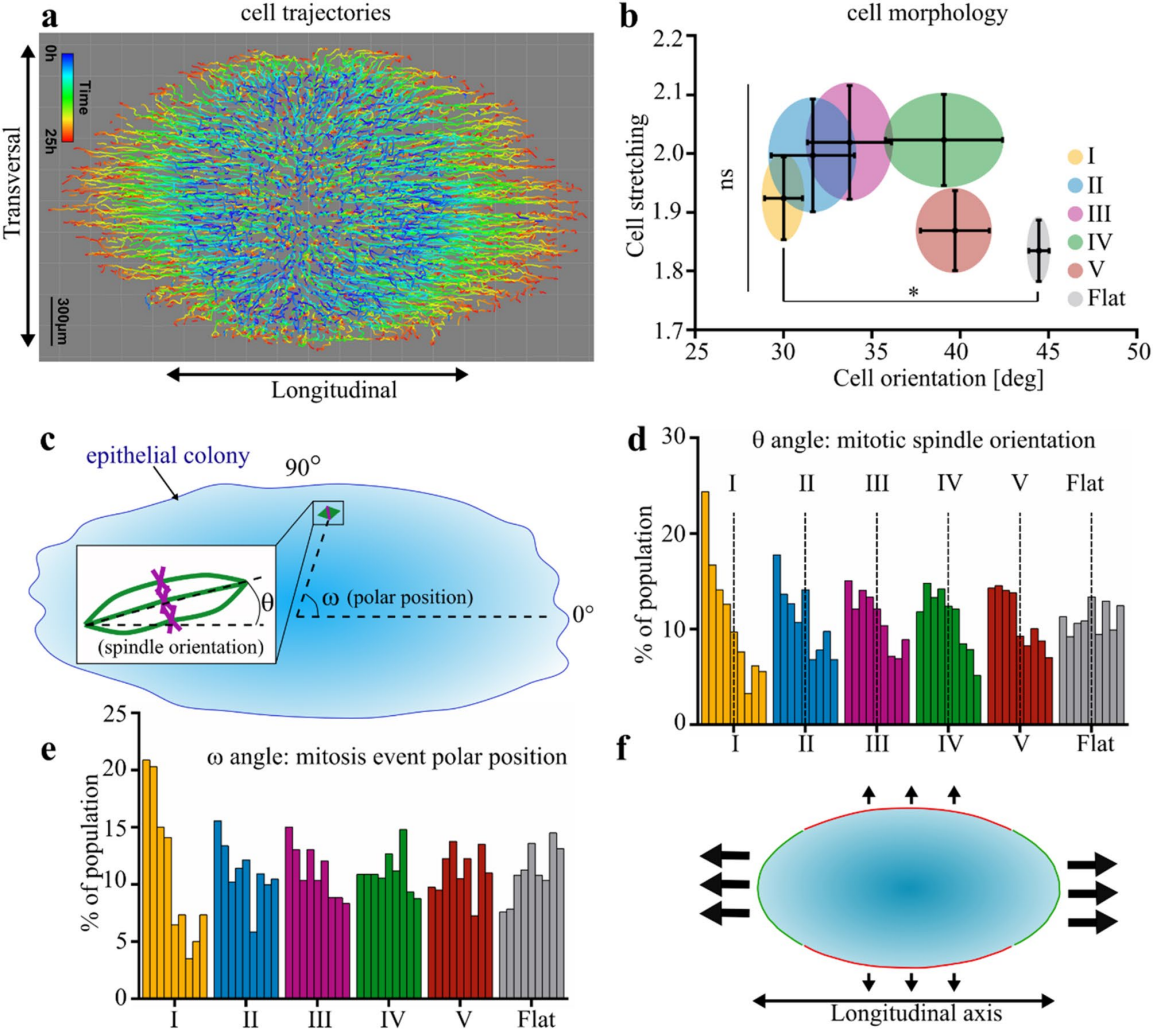
Curvature-dependent orientation of key morphogenetic mechanisms. (**a**) Cell trajectories during 24 h of growth on Topo I (see also Supplementary Movie 1). Note that cells initially starting transversally turns toward the longitudinal axis. (**b**) Average cell stretching (Aspect Ratio) and cell alignment with topography within the epithelial colony (mean + SEM). Five separated fields per colony, four independent colonies per topography. Non parametric Kruskal–Wallis test, *P < 0.05. (**c**) Schematic of the positional parameters quantified for mitosis. θ angle: orientation of the mitotic spindle relatively to the topography longitudinal axis ω angle: angular coordinate of the dividing cell using the longitudinal axis as reference axis and the colony centroid as origin. (**d**) Distribution of the mitotic spindle orientation θ (bin width: 10°) 300–400 mitosis from 4 independent colonies for each topography. The dotted lines separate the angles below 45° (indicating an alignment with the substrate longitudinal axis) and above 45° Kruskal–Wallis non-parametric test shows a statistical difference between medians P < 0.0001. (**e**) Distribution of the angular coordinate ω of cells in mitosis (bin width: 10°), 300–400 mitosis from 4 independent colonies for each topography. Kruskal–Wallis non-parametric test shows a statistical difference between medians P < 0.0001. (**f**) Model of the epithelial anisotropic growth. The surface longitudinal axis is oriented horizontally. Transversally facing colony borders are hampered in a curvature-dependent fashion. Longitudinal orientation of cell migration and cell proliferation sustain the growth of longitudinally facing borders, leading to the anisotropic deformation of the colony.

The growth along a specific direction of space is the end-result of synergistic morphogenetic processes such as cell rearrangement and alignment, cell stretching, collective migration and oriented mitosis^26^. We thus decided to determine which of these morphogenetic mechanisms might be guided by the curvature pattern of the substrate. Among these mechanisms, collective migration has been widely investigated over the years. We tracked the trajectory of cells in the growing colony from Supplementary Movie 1 (Fig. 5A). The migration trajectories initiating along the longitudinal axis remained straight while the trajectories starting in the transversal direction seemed hampered and eventually turned along the longitudinal axis. Consistent with our previous observation on the formation of pluricellular protrusions (Fig. 2) and orientation of the actin cytoskeleton (Figs. 3, 4), this confirmed that curvature sensing leads to a reorientation of cell trajectories within the monolayer resulting in curvature-dependent longitudinal growth of the epithelia.

We then looked at cell morphology (Fig. 5B). Indeed, the anisotropic growth of epithelia during development or post-injury re-epithelialization also includes events of cell stretching and alignment^26^. While we observed a trend toward curvature-dependent cell alignment similar to what we observed for nuclei (Supplementary Fig. S4), we did not manage to obtain statistically significant difference compared to the Flat control (with the exception of Topo I). This is in contrast with the effect of anisotropic topographies on single cells, where cells tend to show a significant increase of elongation^6^. It then highlights that the response of the epithelium to curvature is likely to be a collective phenomenon.

Beside migration and cell morphology, the oriented proliferation is also a key factor of oriented morphogenesis and participate in the elongation of various tissues in vivo^26^. Consistently, we checked whether the cell-scale topography could also reorient cell proliferation. We quantified both the orientation of the mitotic spindle (θ angle) and the angular position of mitosis events using the longitudinal axis of the surface (ω angle) and the colony centroid as references (Fig. 5C). Importantly, the θ angle distribution presented a clear skew toward low angles. This bias got smaller as the curvature decreased until reaching a homogenous distribution on the Flat control (Fig. 5D). The ω angle distribution showed that the majority of mitotic events had an angle below 45° on high curvature topographies (Topo I–III) (Fig. 5E). This means that more cell divisions are occurring in the longitudinally faced sectors of the colony on highly curved topographies, which may contribute to the faster growth of the colony in the longitudinal direction. This interestingly demonstrates a topography-based, curvature-dependent control of oriented proliferation: anisotropic topography leads to a longitudinal division of cells while the magnitude of the local convexity modulates the extent of this effect.

Altogether, we show that periodic curvature exerts a global control over epithelial morphogenesis by targeting various key mechanisms such as migration, cell alignment or proliferation. These effects combined lead to a progressive slowdown of transversal extension compared to longitudinal extension, causing the observed elongation and the associated elliptic shape of the epithelium (Fig. 5F). Importantly, all these effects are proportional to the transversal curvature. It confirms that the transversal curvature is the ruling parameter that modulate the anisotropy of the growth of epithelial monolayers.

## Discussion

The phenomenon of contact guidance leads to the oriented migration or growth of cohesive cell population such as epithelia in presence of anisotropic microenvironment. The majority of studies investigating topographical contact guidance of epithelia used anisotropic and edge-containing surfaces^4,6,7,21,27–29^. The fact that fibrillary ECM such as collagen can guide the collective migration of MDCK island^30^ highlighted the necessity to work with geometries more akin to the in vivo context and has spurred interest into the importance of curvature. Previous works established that epithelial monolayers are sensitive to cell-scale curvature. Some of them used confined geometries such as cylinders or hollow tubes. Confinement either impeded further growth or artificially forced the growth toward a specific direction of space^13,14,17,31^. For example, previously used wires or hollow tubes inherently cause a lateral confinement and restrict the monolayer progression to the cylinder axis. In addition, this lateral confinement modifies the dynamics of the monolayer, causing plugging (hollow tubes)^14^ or altered migration velocity (wires)^13^. By contrast, “open” geometries where every direction of growth in the xy plane is theoretically possible do not constrain the epithelial colonies and preserve their ability of auto-organization. They then allow to investigate the efficiency of cell-scale curvature to guide tissue morphogenesis. A recent report indicate that endothelia grow anisotropically over sinusoidal surfaces suggesting that topographical discontinuities are dispensable and that curvature of the environment can by an efficient morphogenetic cue^18^. However, changes in the sinusoid wavelength between conditions alter the period of curved sections. As a consequence, endothelial islands of similar areas may see less curvature-originated guiding cues when cultivated over long wavelength surface compared to short wavelength surface, which may explain the overall change in anisotropic growth. The importance of the spatial density of topographical pattern had been suggested in pioneering work^2^ and reported for angular, nanometric patterns^19,20^. We eliminated this bias in our system by developing surfaces with different curvatures but with constant period. This is accompanied with a change in ridge width, which could introduce a new bias. However, we do note that Topo I and Topo II have ridges of comparable width and yet induce noticeable differences in all the supercellular and subcellular readouts considered, indicating that curvature is indeed the ruling topographical parameter. With this approach, we demonstrated that the local curvature is a potent cue tuning the anisotropization of epithelium growth. The curvature-driven anisotropic growth is characterized by a curvature-dependent exclusion of nuclei from convex region and alignment of actin stress fiber with the longitudinal axis (Figs. 3, 4). This suggests that areas of convex curvature work as longitudinally oriented topographical barriers hampering tissue growth transversally to them.

Epithelial morphogenesis results from the synergy between various morphogenetic processes such as collective migration, intercellular rearrangement, cellular morphological changes and oriented cell division^26^. Interestingly, we observed a wide effect as both migration and proliferation presented a curvature-dependent spatial bias toward the longitudinal axis of the surface (Fig. 5). This puts the magnitude of anisotropic curvature as a central parameter of the microenvironment able to modulate many aspects of tissue growth and morphogenesis. The question of whether the substrate curvature affects each morphogenetic mechanism individually or rather coordinate them through an unknown mechanism is yet to be determined. To that regard, the spatial bias of pluricellular protrusion formation is interesting (Fig. 2). These are sites of particularly important cell-ECM traction forces^23,32^ and may play a role in local guidance^33^. It then raises the interesting possibility that the spatial anisotropy of pluricellular protrusion leads in turn to a corresponding anisotropy of the monolayer mechanical stress. Given that cell and mitosis orientation are sensitive to the local mechanical stress direction^34–36^, it could then explain the curvature-dependent reorientation of mitosis observed in Fig. 5. To test that hypothesis, it would therefore be very interesting to quantify the intercellular and cell-substrate mechanical stresses to determine the influence of the local substrate curvature on it and the variation of stress magnitude/orientation along the longitudinal vs transversal axis.

Several hypotheses have been formulated to explain the mechanism of transduction of the topographical information. The Curtis and Clark hypothesis suggests a role for topographical discontinuities (edges) in topography sensing^2^ for example through the accumulation of Focal Adhesions along the edges^10^. Alternatively, O’Hara and Buck hypothesized that the lower parts (grooves) of nanometric topography were out of cells’ reach thereby restricting focal adhesions to the ridges^37^. With our curved, cell-scale topographies, these hypotheses do not hold. First because our topographies are smooth and edgeless and second because their dimensions allow for cell-substrate contact at every point, even in the grooves (Fig. 1A). Besides, we did not observe dominant and repetitive pattern of focal adhesions (data not shown). We thus looked for other cellular components that might be involved in the sensing of curved surfaces. We hypothesized that eligible candidates should exhibit an anisotropic organization reflecting the anisotropy of the substrate and being proportional to the curvature. Both nuclei distribution and F-actin orientation satisfy these criteria. Several studies establish nuclei as essential players of cell reaction to topography^38^. We demonstrated that single cells avoid crossing convex regions owing to the tendency of nuclei to slide away from convex curvature^12^. This is consistent with the low nuclei density over convex regions in our settings indicating that the transversal crossing of convex regions is impaired. In addition, the orientation of actin stress fibers have been shown to depend on the local curvature^39,40^. While no mechanism has been fully demonstrated, a model suggests that the final orientation of stress fibers results from the balance between the bending energy cost and the cell contractility^41^. According to this model, cells on a low convex curvature will orientate their stress fibers along the direction of maximum curvature to compensate the deformation resulting from cell contractility. We made observations consistent with this model as we indeed observed transversal stress fibers on our topographies with low convex curvature radii, Topo IV and V (22 and 44 μm, respectively see Fig. 4 and Supplementary Fig. S3). This finding is consistent with the available literature, where epithelial cells growing over cylindrical substrates of 16–40 μm curvature radius also present transversally oriented stress fibers, e.g. perpendicular to the cylinder axis^13,39^. On higher convex curvature, the logic behind the model of Biton and Safran would then suggest an orientation of actin stress fiber along the path of minimum curvature to compensate for the rising energetic cost of actin bending as the substrate curvature is increased. We can then understand why convex regions seem to work as “topographical barriers” on our high curvature topographies (radii below 10 μm). In that case, actin stress fibers tend to follow in the form of longitudinal bundles rather than to cross. Together, nuclei exclusion from, and actin reorientation along convex regions would then result in a spatial bias of various morphogenetic mechanisms such as collective migration or proliferation along the longitudinal surface axis.

Our surfaces also contain concave regions, specifically at the base of the slopes between the grooves regions and the ridges. This concave curvature also repeats itself all the way along the longitudinal axis. Epithelial monolayers cultured in concave grooves or hollow tubes orientate their actin along the longitudinal axis^14,42^. It is thus possible that concave curvatures also play a role in the topography-controlled directional growth of our epithelial colonies. However, we only observed curvature-dependent nuclei exclusion from convex areas (Fig. 4). In addition, we observed a drop of actin density right at the point of maximal convex, but not concave, curvature (Fig. 3). Although we cannot rule out a role of concave curvature in the contact guidance of growing epithelia, we estimate that the convex curvature is the driving geometrical cue in our experimental setting.

The notion of contact guidance had been applied to curved surfaces presenting either lateral confinement or varying topographical spatial densities. Here, we achieved the adjustable anisotropization of epithelial growth using smooth grooves and ridges with various degrees of curvature but equal periods and depths. In this work, we thus demonstrate that the magnitude of curvature is intrinsically a potent and tunable guiding cue for the morphogenesis of epithelia. Longitudinally oriented convex regions act as “topographical barriers” whose “strength” depends on the curvature magnitude. Repetitive encounter between the growing epithelium and these barriers progressively reorient the cytoskeleton, the cell migration and proliferation and leads to the whole epithelium elongation. As curvature is a geometric feature that can take an infinity of values, it opens interesting possibilities of fine-tuning the tissue behavior by substrate design. Growing epithelia may encounter curved and anisotropic landscape in vivo for example in the vicinity of blood vessels or thick collagen bundles of the dermis^43^. In addition, tissue curvature has a strong influence of the outcome of cancer morphogenesis^44^. Thus, we suggest that the control of epithelial anisotropic growth through local curvature participate in tissue morphogenesis in vivo, together with classic chemical or mechanical cues. The ability of cell-scale curvature to induce and modulate the anisotropic growth of epithelial sheets bears a great interest for the design of in vitro implant device aiming at fostering the growth of epithelial tissues.

## Material and methods

### Preparation PDMS surface

Five steel master surfaces were micro-fabricated. They consisted of an array of parallel grooves with a period of 100 µm and an amplitude of 10 µm. For the five surfaces the mid height valley width equals the mid height peak width. Each surface presents a distinct peak curvature ranging from a straight edge (Topo I) to a sinusoidal curve (Topo V). The surfaces were microstructured on 316 L stainless steel coins (diameter 15 mm, thickness 1.5 mm) using a two-step electrochemical process. The raw materials were first mechanically polished to obtain a mirror finish before being spin-coated with a polymeric resin (10 µm thick). The first process step mask pattern (diameter 8 mm) was then created through local UV laser ablation of the resin coating. Mass transport-limited electrochemical dissolution was then performed under optimized hydrodynamic conditions. The experiment was stopped when a precise electrical charge corresponding to the desired dissolution depth was passed through the system. For the second process step, the remaining polymeric mask was laser ablated within a 10 mm diameter and a second electrochemical dissolution step was applied until the final topography was reached. The geometric parameters of the first step mask and the electrical stop charges of both electrochemical steps were obtained by numerical simulations (custom Labview 2D Laplace equation solver using a boundary elements method).

Plastic replicates were then produced by hot embossing of the steel masters on 35 mm Petri dishes. The final PDMS surface (thus with the same topography as the steel master templates) were produced by pouring liquid PDMS (Sylgard 184, 1 part of curing agent for 10 part of PDMS) into the plastic replicate and incubated 6 h at 80 °C.

### Estimation of local curvature of PDMS surface

Fibronectin-coated PDMS surfaces were immunostained. Alternatively, rhodamine-labelled fluorescent fibronectin (20 μg mL^−1^, Cytoskeleton Inc.) was used. The transversal cross-section of the topography was obtained from reslicing of a vertical confocal z stack (z step 0.37 μm, 60 × lense of AN 1.4 yelding an optical slice of 1 μm) and subsequent binarizing and skeletonizing the fibronectin signal (Supplementary Fig. S1A). An equation $y=f(x)$ was fitted to the cross-section by non-linear regression to describe the surface altitude as function of position along the transversal axis (Supplementary Fig. S1B). The curvature κ was then obtained at each point by $k = \frac{{\ddot{y}}}{{\left( {1 + \dot{y}^{2} } \right)^{{\frac{3}{2}}} }}$ where the dot and double dot represent the first and second derivative of *f*(*x*), respectively. The local maxima and minima of κ over a single period of the topographical pattern correspond to the most convex and most concave points, respectively. Resulting values are shown in Supplementary Fig. S1C,E. Color-coded representations (Supplementary Fig. S1D) of the local curvature were produced by averaging κ between surfaces at each points and using binarized fibronectin staining as mask.

### Cell culture

The parental MDCK cell line (canine epithelial kidney) was obtained from the Cell Bank of the State of Rio de Janeiro (BCRJ, cell code 0168). MDCK were cultured in Dulbecco’s Modified Eagle Medium (Sigma D6046) low glucose, supplemented with 10% Fetal Bovine Serum (Gibco, lot #210415K), MEM Non-essential Amino Acid (Sigma, M7145), L-glutamine 2 mM (Sigma, G7513). For the growth of epithelial colonies over PDMS substrates, PDMS substrates are sterilized with ethanol and coated with bovine plasma fibronectin (ThermoFischer Scientific, 33010018 20 μg mL^−1^) for 1 h at room temperature. In parallel, small PDMS blocks with a central, custom-made well are first sterilized and passivated with a 5% solution of BSA in water and then a 5% solution of Pluronic acid F-127 (Sigma, P2443) and placed over the PDMS substrate. A MDCK suspension (1.15 million cells mL^−1^) is loaded into the wells incubated overnight before carefully removing the wells.

### Antibodies and reagents

Antibodies used are available in Supplementary Informations (Supplementary Note 2). Other staining reagents are as follows: Alexa488-phalloidin (ThermoFischer Scientific, A12379 1:200), Alexa568-phalloidin (ThermoFischer Scientific, A12380 1:200), Alexa647-phalloidin (ThermoFischer Scientific, A22287 1:200), Hoechst 33342 (ThermoFischer Scientific, H1399, 1:1,000).

### Live cell imaging

For live cell imaging, cells adhering onto the topographies where loaded with the dye CellTracker Red CMPTX (ThermoFischer) and placed in an incubation chamber (Okolab) at 37 °C, 5%CO_2_ and H_2_O saturated. Z-stacks were acquired at regular time intervals with a 20 × magnification objective. Particle tracking was done on vertical maximum projection of the z-stacks using Imaris software (Bitplane).

### Immunofluorescence

For immunofluorescence, the samples are washed with warm PBS with Calcium and Magnesium and fixed and permeabilized for 15 min at room temperature (formaldehyde 4%, Triton X-100 0.2% in PBS with Calcium and Magnesium). The samples are then rinsed blocked for 30 min (5% BSA, 0.2% Triton X-100 and 5% donkey serum in TBS). The samples are then rinsed and incubated with the primary (1 h) then secondary (45 min) antibody with 3 washes in between in staining buffer (1% BSA and 0.2% Triton X-100 in TBS). The samples are then rinsed 5 times and mounted in glycerol 90%, 0.5% *N*-propyl gallate (Sigma 02370). Images are acquired on a Olympus IX71 epifluorescence microscope, a Zeiss Axiozoom V.16, or a Leica TCS-SPE Confocal microscope.

### Actin orientation

To determine the orientation of F-actin in the xy plane, z-stack from confocal microscopy images were analyzed slice by slice with the OrientationJ ImageJ plugin^25^. The dominant orientation was averaged between slices of a given stack then between stacks of a given epithelial colony to yield the mean F-actin orientation of the colony. Six stacks by colonies were used.

### Analysis of mitosis

To analyze the position parameters of mitosis event, an image of the entire epithelial colony stained for nuclei and tubulin was obtained using an Axiozoom V.16. The orientation of mitosis was determined based on the orientation of the mitotic spindles. The polar coordinate was manually determined using the longitudinal axis and the colony centroid as references.

### Analysis of nuclei distribution and deformation

The average number of nuclei positioned over the points of highest convex or concave curvature was quantified by counting each nuclei overlapping these points from transversal section of confocal z-stacks and subsequent normalization by the number of nuclei overlapping an arbitrary point localized on a flat section of the topography. To estimate the topography-induced deformation of nuclei, nuclei shape from transversal cross-sections of z-stacks were binarized. The solidity (ratio between the area of the object and its bounding ellipse) of the resulting shapes was obtained using ImageJ.

### F-actin and nuclei density maps

For nuclei, individual fields of view of nuclei were binarized to a pixel value of 1 for nuclei and 0 for background and carefully adjusted by translation between them to match their underlying topographies. The aligned and superposed projections are then subjected to an average projection. Therefore, each pixel value represents the average number of nuclei overlapping that particular point of the topography. For F-actin, sum projections of individual stacks were obtained and each normalized by its fluorescence mean. Then, all these projections were aligned together by translation, and averaged together, yielding an average F-actin density map.

### Morphological analysis of colony

Images of the entire colonies were obtained after two days of growth. The colony alignment was calculated as the deviation angle between the topography longitudinal axis and the major axis of the fitting ellipse of the colony. The colony aspect ratio is the ratio between the longer and shorter axis of the fitting ellipse. The edge velocity was performed using the plugin QuimP for ImageJ^45^. Shapes of individual cells were determined by manual outlining and used to calculate the cell orientation and elongation.

### Pluricellular protrusion formation (tortuosity)

Spatial bias in the formation of migration fingers was quantified through a “Protrusion Bias Index”. Details on the index are available in Supplementary Fig. S2 and Supplementary Note 1.

### Statistical analysis

All the statistical tests (parametric and non-parametric) were performed using GraphPad Prism 8.

## Supplementary information

Supplementary Information.

Supplementary Video 1.

Supplementary Video 2.

Supplementary Video 3.
