# Supplementary material for: Topographical curvature is sufficient to control epithelium elongation
Source: Sci Rep. 2020 Sep 8;10:14784. doi: 10.1038/s41598-020-70907-0 (PMC7479112; doi:10.1038/s41598-020-70907-0)
Supplement: Supplementary file 1 — Supplementary Information. [file 41598_2020_70907_MOESM1_ESM.docx]

**Topographical curvature is sufficient to control epithelium elongation**

*Pablo Rougerie^*1^, Laurent Pieuchot***^2^, Rafaela Silva dos Santos^1,3^, Julie Marteau^4^, Maxence Bigerelle^4^, Pierre-François Chauvy^5^, Marcos Farina^1^, Karine Anselme^2^*

**Supplementary Data**


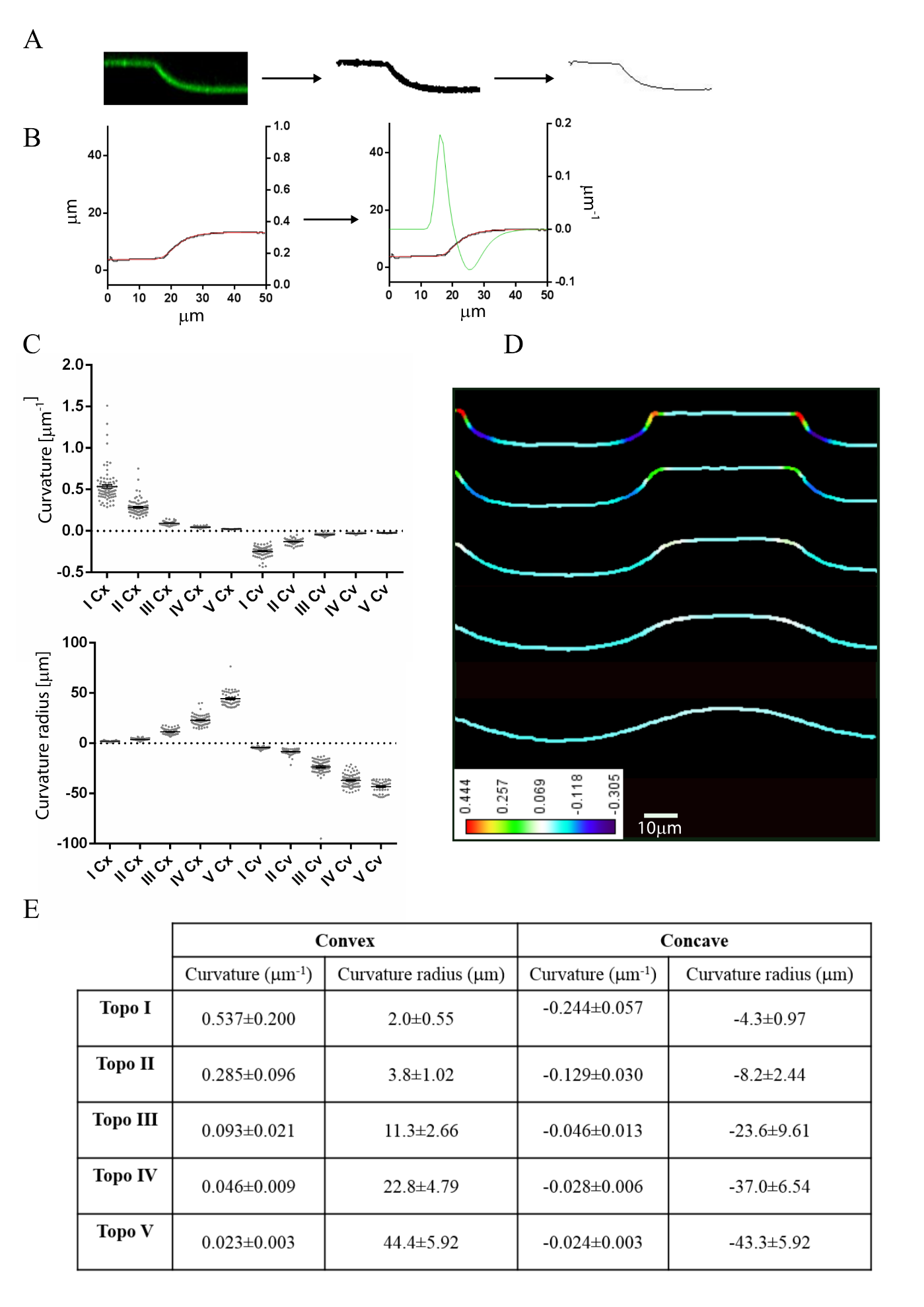


**Supplementary Figure S1: Quantification of the surface curvature.**

The quantification was performed on the final PDMS replicates used for cell culture.

**(A)** The fibronectin staining from transversal cross section is thresholded and skeletonized to obtain the outline of the topography. **(B)** An equation (red curve) is then fitted to the topography outline (black curve) by non-linear regression. Note that on the plot the topography is showed upside-down. The curvature at each point of the fitted equation is then calculated (green curve). **(C)** The quantification is performed from 27 cross-sections obtained from 3 independent PDMS replicates for each topographies. The maximum (convex, noted Cx) and minimum (concave, noted Cv) curvatures and corresponding radii of curvature for each cross-section are showed as scattered dot plot (mean + SEM shown in black). **(D)** color-coded curvature of the topography transversal section. Note the peak of positive (convex) curvature at the side of the ridge (topo I, II, III, IV) and at the apex of topo V. **(E)** Table of the average curvatures and radii of curvature (plus/minus standard deviation). The values are obtained as described in the Material and Methods and as illustrated in FigS1A-B and are presented graphically in Figure S1C.


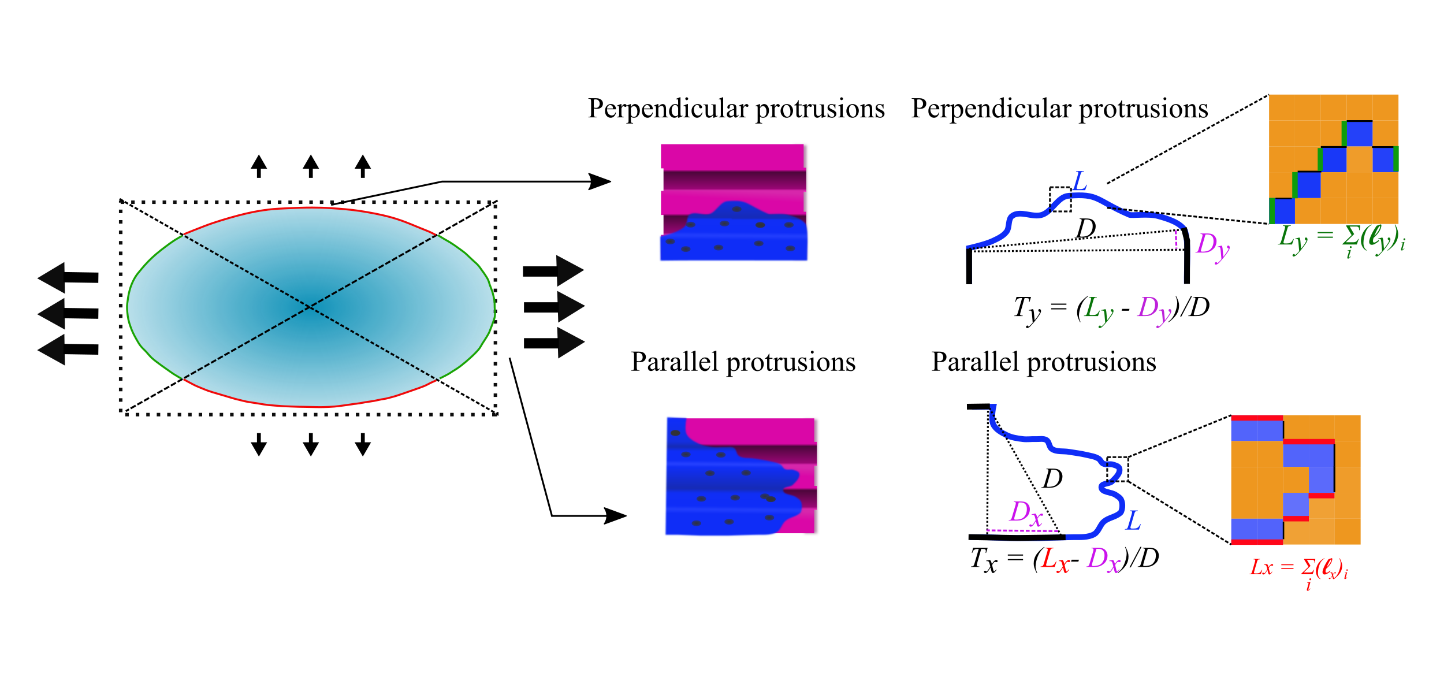


**Supplementary figure S2: curvature-dependent alignment of F-actin with the surface longitudinal axis**

Schematics of the calculation of the Protrusion Bias Index (see Supplementary Note 1 for details). *Left*: the expanding monolayer is divided in four sectors East, West, North and South defined by the 2 diagonals of the fitting rectangle. Borders in each sector tend to grow radially outward. *Right*: At the North and South borders, the tendency of the monolayer to form migration fingers extending transversally to the topography is calculated by the transversal tortuosity *T_y_*. At the East and West migration fronts, the tendency of the monolayer to form migration fingers longitudinal to the topography is calculated by the longitudinal tortuosity *T_x_*. The protrusion Bias Index is then taken as the contrast between the longitudinal tortuosity *T_x_* at the East and West borders and the transversal tortuosity *T_y_* at the North and South: $= \frac{T_{x,EW}-T_{y,NS}}{T_{x,EW}+T_{y,NS}}$ . A value of zero indicates an isotropic condition where migration fingers grow similarly on all borders. A positive value indicates a spatial bias in migration fingers formation: longitudinal growth is favored at the East and West borders whereas transversal growth is hampered at the North and South borders, resulting in anisotropic growth of the epithelial colony.


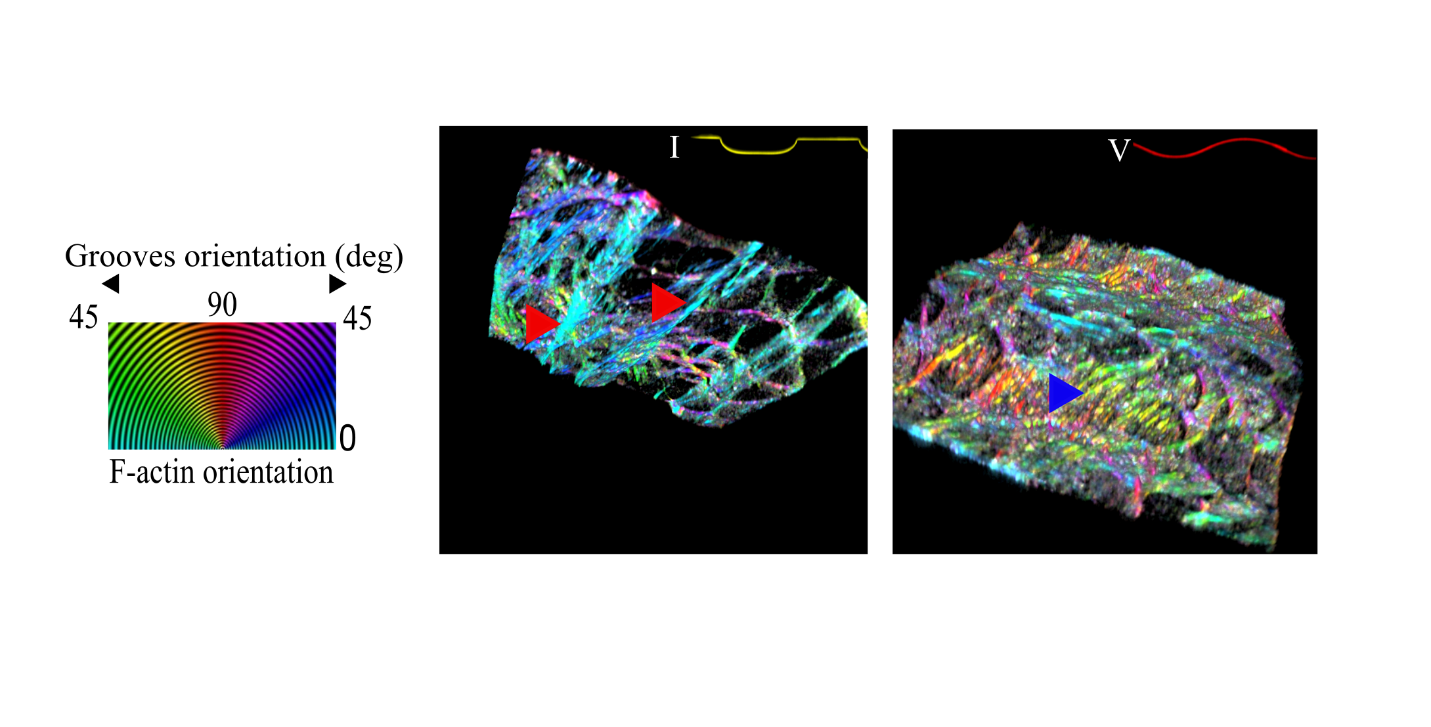


**Supplementary Figure S3: Orientation of actin stress fibers depends on the substrate curvature**

Left: color coding of actin orientation. Zero degrees corresponds to the longitudinal axis of the topography. Middle: 3D reconstruction of a confocal z-stack of an epithelial monolayer growing on Topo I (see Movie 2). Thick bundles of longitudinal actin can be seen in the vicinity of the most convex region, without crossing it (red arrowheads). Right: 3D reconstruction of a confocal z-stack of an epithelial monolayer growing on Topo I (see Movie 3). Transversal actin fibers can be seen across the convex regions (blue arrowheads).


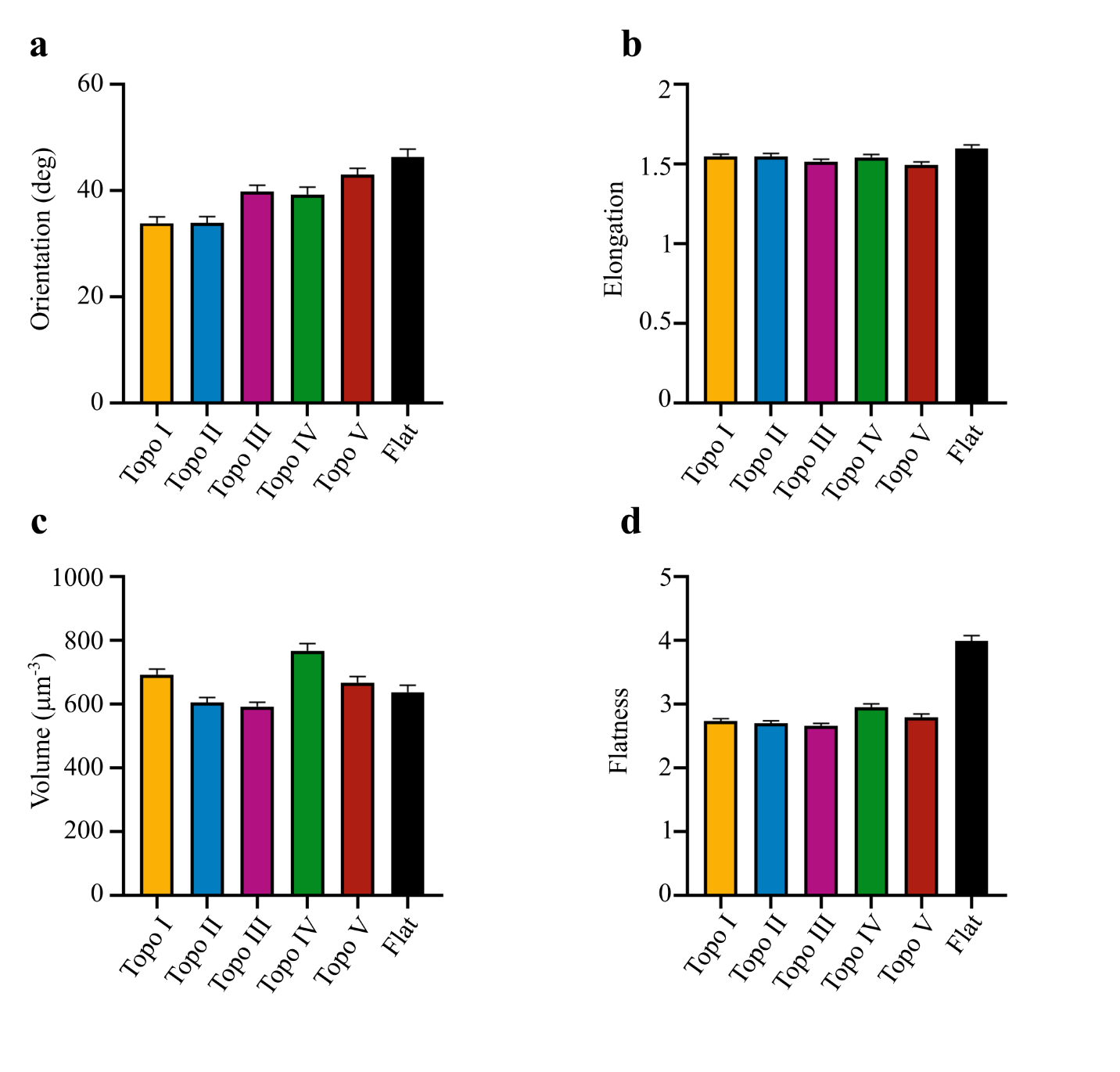


Supplementary Figure S4: Morphology and orientation of nuclei over the different topographies

The morphological parameters of nuclei were obtained by three-dimensional segmentation of reconstituted vertical stacks of nuclei from epithelial colonies grown over the different topographies. a. the orientation of nuclei is shown relatively to the longitudinal axis of the topography: Zero degree represents a perfect alignment of the nucleus. b. Elongation of the fitting three-dimensional ellipsoid of the nuclei, defined by the ratio between the longest axis and the medium axis of the ellipsoid. c. volume of the fitting three-dimensional ellipsoid of the nuclei. d. flatness of the fitting three-dimensional ellipsoid of the nuclei, defined by the ratio between the longest and shortest axis of the ellipsoid. Average ± SEM of 380 to 500 nuclei obtained from 3 independent experiments (3 separate fields per experiment).

**Movie 1: anisotropic growth of an MDCK epithelial colony over Topo I**

The round epithelial colony is loaded with the cell dye CMPTX and its growth is monitored during 24 hours by confocal microscopy. Note that in the movie the longitudinal axis of the topography is oriented vertically. Pixel tracking showing the trajectories of cell is indicated.

**Movie 2: 3D reconstruction of the actin cytoskeleton from an epithelium growing on Topo I**

The F-actin is color-coded for its orientation relatively to the longitudinal axis of the surface as indicated in Figure 3D and Supplementary Figure S2B. Thick bundle of stress fibers (appearing in cyan) runs longitudinally along the region of highest convex curvature, without crossing it.

**Movie 3: 3D reconstitution of the actin cytoskeleton from an epithelium growing on Topo V**

The F-actin is color-coded for its orientation relatively to the longitudinal axis of the surface as indicated in Figure 3D and Supplementary Figure S2B. No longitudinal stress fibers are observed. By contrast, transversal stress fibers can be seen across the convex regions (see 4. Discussion for explanation).

**Supplementary Note 1: quantification of the spatial bias in migration finger formation**

The Protrusion Bias Index is computed for each sector using a custom written ImageJ macro. An image of the entire epithelial colony is obtained by fluorescence microscopy using a stereomicroscope. The colony is then divided in four sectors North, South, East and West using the diagonals of the bounding rectangle. The colony border is then manually outlined in each sector. Given that the colony has an elliptical shape, computing the tortuosity using the whole border would introduce a bias due the overall curved shape of the border. The colony outline of each sector is thus further divided in 8 parts to detect only the local tortuosity due to the presence of migration fingers. On an isotropic substrate, migration fingers are expected to grow radially. In other words, migration fingers of the Northern sector would point northward on average, migration fingers of the Western sector would point westward on average, etc. A bias in the formation of protrusion fingers toward the longitudinal axis of the topography would then result in a favored formation of westward or eastward migration fingers in the Western and Eastern sectors and a diminished formation of northward or southward migration fingers in the Northern and Southern sectors. To quantify that, the longitudinal tortuosity *T_x_* is calculated for the western and eastern border and the transversal tortuosity *T_y_* is calculated for the northern and southern borders. The longitudinal (respectively, transversal) tortuosity is the total length covered along the longitudinal (respectively, transversal) axis by a given section of the colony border divided by the distance in straight line between the two extremities of that section as illustrated in Supplementary Figure S2). To represent the discrepancy between the average longitudinal tortuosity $\bar{T_{x}}$ calculated in the Western and Eastern sectors and the transversal tortuosity $\bar{T_{y}}$ calculated in the Northern and Southern sectors, we computed the final Protrusion Bias Index as the contrast between them: $\frac{\bar{T_{x}}- \bar{T_{y}}}{\bar{T_{x}}+\bar{T_{y}}}$. When the epithelial colony forms radially symmetric migration fingers (for example on a flat, isotropic surface) the index will have a value of zero. When the formation of migration fingers is favored in the Western or Eastern sectors relatively to the Northern or Southern sector, the Index will yield a positive value.

**Supplementary Note 2: antibodies**

monoclonal mouse anti-ZO1 (ZO1-A12, ThermoFischer Scientific, 33-9100, 1:100),

mouse anti-Tubulin (DM1A, Sigma, T9026, 1:300)

rabbit anti-Fibronectin (Abcam, ab2413, 1:200);

secondary antibody donkey anti-Mouse Alexa488 (ThermoFischer Scientific, A21202, 1:300),

secondary antibody donkey anti-Mouse Alexa568 (ThermoFischer Scientific, A10037, 1:300),

secondary antibody donkey anti-Mouse Alexa647 (ThermoFischer Scientific, A31575, 1:300),

secondary antibody donkey anti-Rabbit Alexa 488 (ThermoFischer Scientific, A21206, 1:300),

secondary antibody donkey anti-Rabbit Alexa 568 (ThermoFischer Scientific, A12380, 1:300),

secondary antibody donkey anti-Rabbit Alexa-647 (ThermoFischer Scientific, A31573 1:300)
